# Gastrostomy as an Adjunctive Gastropexy in Recurrent Congenital Diaphragmatic Hernia with Repeated Gastric Herniation: A Case Report

**DOI:** 10.70352/scrj.cr.26-0493

**Published:** 2026-10-28

**Authors:** Minori Ishii, Saya Azuma, Yuka Kumata, Yuki Meya, Keita Motoki, Haru Matsumoto, Saki Sugeno, Satsuki Suda, Aiko Aoyama, Toshio Okamoto, Ken Nagaya, Hisayuki Miyagi

**Affiliations:** 1 Division of Pediatric Surgery, Department of Surgery, Asahikawa Medical University, Asahikawa, Hokkaido, Japan; 2 Division of Neonatology, Center for Maternal and Infant Care, Asahikawa Medical University Hospital, Asahikawa, Hokkaido, Japan

**Keywords:** congenital diaphragmatic hernia, recurrence, gastric herniation, gastrostomy, gastropexy, gastric volvulus

## Abstract

**INTRODUCTION:**

Although the survival of patients with congenital diaphragmatic hernia (CDH) has improved with advances in perinatal management, postoperative recurrence remains a significant complication. Risk factors for recurrence include large diaphragmatic defects, patch repair, liver herniation, and minimally invasive surgery. Recurrent CDH is associated with a high rate of subsequent recurrence, and the optimal surgical strategy has not been established. We report an infant with recurrent CDH and repeated gastric herniation who was treated with additional diaphragmatic reconstruction combined with gastrostomy fixation.

**CASE PRESENTATION:**

A male infant with prenatally diagnosed left-sided CDH underwent primary repair on day 4 of life. A grade C defect with medial diaphragmatic deficiency was repaired using a cone-shaped GORE-TEX patch. At 4 months of age, follow-up imaging demonstrated recurrence with herniation of the stomach and colon into the left thoracic cavity. At reoperation, the lateral fixation of the patch remained intact, while its medial, anterior, and posterior portions had detached, and the patch had become displaced laterally. The defect was reconstructed using an additional patch. One week later, he developed a second recurrence accompanied by progressive intrathoracic gastric herniation with mesenteroaxial gastric volvulus, resulting in poor weight gain. At 5 months of age, a fourth operation revealed a similar pattern of patch detachment, with herniation of the stomach and colon. Additional patch reconstruction and gastrostomy fixation were performed. Postoperatively, the patient’s oral intake and weight gain improved markedly, without gastrostomy-related complications. The gastrostomy button was removed at 1 year of age. At 18 months of age (13 months after the final operation and 6 months after gastrostomy removal), the patient demonstrated normal growth and development without recurrence.

**CONCLUSIONS:**

In this patient, the absence of reliable medial anchoring tissue, limitations of patch fixation, and repeated gastric migration may all have contributed to recurrent diaphragmatic reconstruction failures. Although the independent effect of gastrostomy fixation cannot be determined because it was performed concurrently with additional patch reconstruction, it may have helped limit gastric mobility as an adjunct to diaphragmatic re-reconstruction. This combined approach may be considered in selected patients with recurrent CDH, medial diaphragmatic deficiency, and repeated gastric herniation.

## Abbreviation

CDH
: congenital diaphragmatic hernia

## INTRODUCTION

Although the prognosis of CDH has improved with advances in perinatal management, postoperative recurrence remains a significant clinical problem, with reported recurrence rates ranging from 5% to 65%.^1–3)^ Reported risk factors for CDH recurrence include liver herniation, large diaphragmatic defects classified as grades C or D, patch repair, and minimally invasive surgery.^2,4,5)^ Furthermore, recurrent CDH is associated with a high rate of subsequent recurrence, ranging from 25% to 50%, and repeated recurrence is not uncommon.^2,6,7)^

Surgical management of recurrent CDH is often challenging, and there is no consensus regarding the optimal surgical approach.^2)^ Herein, we describe the case of an infant with recurrent CDH and repeated gastric herniation who underwent additional diaphragmatic reconstruction combined with gastrostomy fixation.

## CASE PRESENTATION

A male infant was prenatally diagnosed with left-sided CDH at 24 weeks of gestation. Fetal ultrasonography and MRI performed shortly before delivery demonstrated a non-liver-up CDH with an observed-to-expected lung-to-head ratio of 32%, indicating moderate pulmonary hypoplasia. He was delivered by cesarean section at 37 weeks and 3 days of gestation with a birth weight of 3516g.

After stabilization of his respiratory and hemodynamic status, definitive repair of the CDH was performed through a 5-cm left subcostal oblique incision at 96 hours of life (**Fig. 1**). Intraoperatively, the left lateral segment of the liver, spleen, stomach, colon, and small intestine were herniated into the left thoracic cavity (**Fig. 2A**). The diaphragmatic defect measured 5 × 5 cm. The lateral, posterior, and medial diaphragmatic rims were deficient, and the defect was classified as grade C in accordance with the Congenital Diaphragmatic Hernia Study Group classification^8)^ (**Fig. 2B**). A 7.5 × 7.5-cm, 1-mm-thick GORE-TEX Soft Tissue Patch (W. L. Gore & Associates, Flagstaff, AZ, USA) was used to repair the diaphragmatic defect. An incision was made in the patch, which was then fashioned into a cone-shaped configuration. This configuration was selected to achieve tension-free reconstruction and increase the available abdominal capacity. The patch was secured with interrupted 2-0 braided nylon sutures to the anterior and lateral diaphragmatic rims and to the periosteum of the ribs posteriorly (**Fig. 3**). Medially, there was no diaphragmatic rim present around the esophagus or mediastinum (**Fig. 2B**). Although the parietal pleura remained in this area, it was too thin to provide reliable anchoring tissue, and suturing it was considered to carry a risk of injury to the adjacent organs. Therefore, the medial edge of the patch was placed over the defect with an overlap of approximately 1 cm, but it was not sutured in this area around the esophagus and mediastinum.

**Fig. 1:**
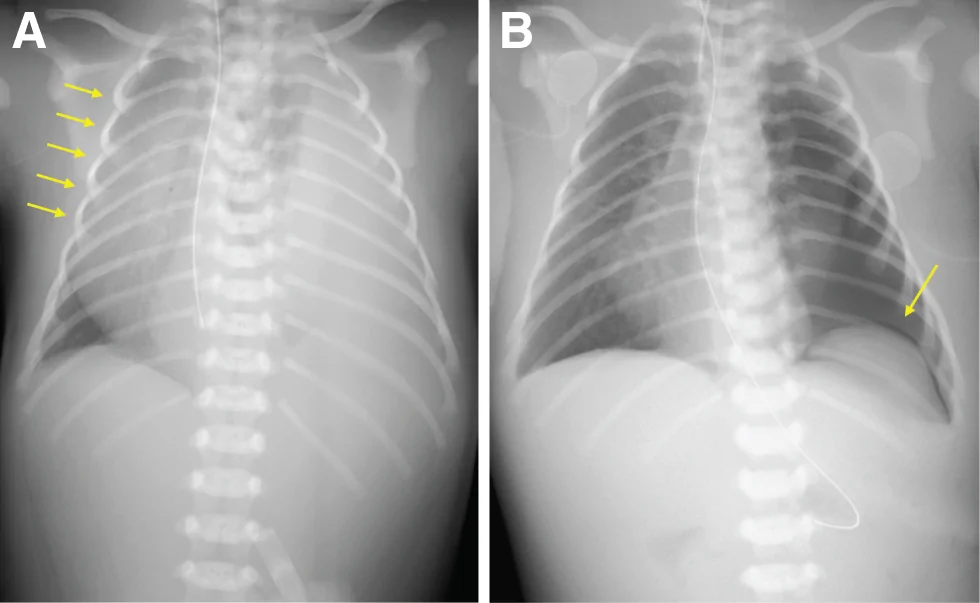
Abdominal and chest radiographs. (**A**) Radiographs obtained shortly after birth show left-sided CDH with rightward mediastinal shift (arrows). (**B**) Radiographs obtained on POD 1 show reconstruction of a dome-shaped diaphragm using a cone-shaped GORE-TEX patch (arrow). CDH, congenital diaphragmatic hernia

**Fig. 2:**
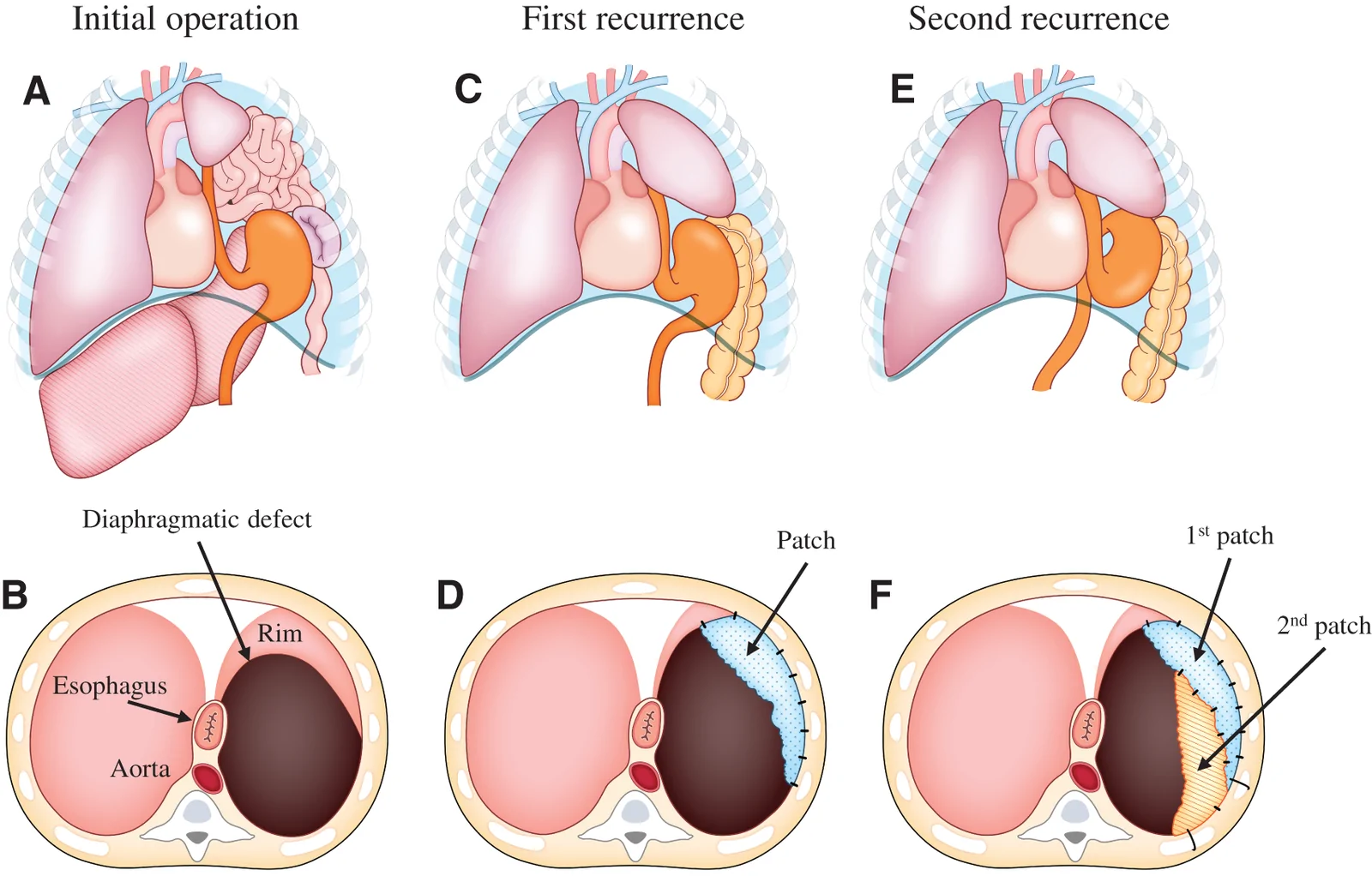
Anatomical findings and patch detachment patterns at the initial operation, first recurrence, and second recurrence. (**A**) In the initial operation, the stomach, spleen, left lateral liver segment, small intestine, and colon were herniated into the left thoracic cavity, with a rightward mediastinal shift. (**B**) Axial schematic view at the level of the diaphragmatic defect during the initial operation. The medial, posterior, and lateral diaphragmatic rims were deficient, and the defect was classified as grade C in accordance with the Congenital Diaphragmatic Hernia Study Group classification. No diaphragmatic rim was present around the esophagus or mediastinum medially. (**C**) At the first recurrence at 4 months of age, the stomach and colon were herniated into the left thoracic cavity. (**D**) Axial schematic view at the first recurrence. The lateral fixation of the original patch remained intact, while the medial, anterior, and posterior portions had detached. The detached patch had rolled up and become displaced laterally. (**E**) At the second recurrence at 5 months of age, the entire stomach and colon were again herniated into the left thoracic cavity. The stomach exhibited mesenteroaxial volvulus, with rotation around its short axis. (**F**) Axial schematic view at the second recurrence. The lateral fixation of the previously placed patches remained intact, whereas the medial, anterior, and posterior portions had detached. The detached patches had become firm and had rolled up toward the lateral aspect. CDH, congenital diaphragmatic hernia

**Fig. 3:**
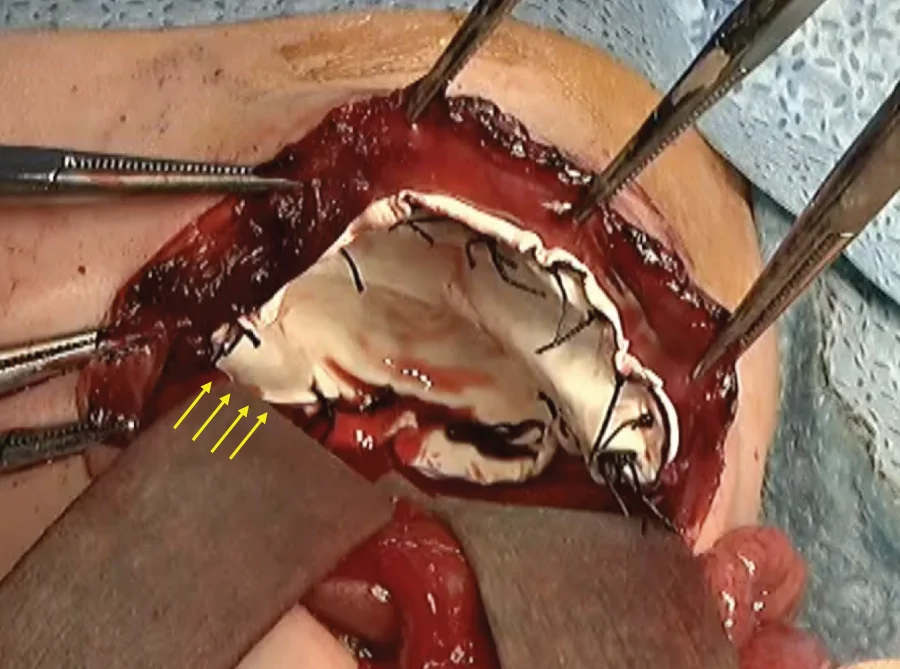
Intraoperative view of the initial diaphragmatic reconstruction. A cone-shaped GORE-TEX patch was secured with interrupted 2-0 braided nylon sutures to the anterior and lateral diaphragmatic rims and to the periosteum of the ribs posteriorly. The medial edge of the patch overlapped the defect by approximately 1 cm but was not sutured because there was no reliable anchoring tissue present around the esophagus or mediastinum. The arrows indicate the unsecured medial overlap.

On POD 20, shortly after extubation, the patient developed a left pneumothorax. Chest radiography raised the suspicion of recurrent CDH; however, exploratory laparotomy confirmed that the diaphragmatic repair remained intact. Fundoplication was performed to manage gastroesophageal reflux.

At 4 months of age, routine follow-up imaging demonstrated herniation of the stomach and colon into the left thoracic cavity, leading to a diagnosis of recurrent CDH (**Fig. 2C**). At reoperation, the diaphragmatic defect measured 5 × 3.5 cm. The lateral sutures of the original patch remained intact; however, the patch was no longer present along the anterior, posterior, or medial aspects of the defect and had rolled up and become firm at the lateral aspect (**Fig. 2D**). No definitive point of initial disruption could be identified. However, the lateral displacement and rolled configuration of the patch suggested that detachment may have begun medially and subsequently progressed anteriorly and posteriorly.

Adhesions associated with the previous fundoplication limited dissection around the gastric cardia and esophagus and further complicated reconstruction of the medial aspect of the defect. The adhesions at the gastric cardia were left undisturbed to avoid injury, while the gastric body was reduced into the abdominal cavity. The laterally displaced original patch was preserved. A 7 × 5-cm, 1-mm-thick GORE-TEX Soft Tissue Patch was fashioned into a cone-shaped configuration and secured with interrupted 2-0 braided nylon sutures (Surgilon; Covidien, Mansfield, MA, USA). The additional patch was anchored to the residual diaphragmatic rim anteriorly, directly to the ribs posteriorly, and to the preserved original patch laterally. As with the initial repair, the medial edge was placed over the defect with an overlap of approximately 1 cm but was not sutured because there was no reliable medial anchoring tissue available.

One week later, the stomach again herniated into the left thoracic cavity (**Fig. 4**). Progressive intrathoracic migration of the stomach with mesenteroaxial gastric volvulus resulted in poor weight gain, and a fourth operation was performed at 5 months of age. Intraoperatively, the recurrent defect measured 5 × 3 cm, with herniation of the entire stomach and colon (**Fig. 2E**). As observed at the previous reoperation, the lateral fixation of the patch remained intact, whereas its medial, anterior, and posterior portions had detached (**Fig. 2F**). The previously placed patches were preserved. A 10 × 6-cm, 1-mm-thick GORE-TEX Soft Tissue Patch was fashioned into a cone-shaped configuration and secured with interrupted 2-0 braided nylon sutures to the existing patch laterally. The additional patch was anchored to the residual diaphragmatic rim anteriorly and directly to the ribs posteriorly. Medially, because no reliable anchoring tissue was present around the esophagus or mediastinum, the patch was placed over the defect with an overlap of approximately 1 cm but was not sutured. Gastrostomy fixation was then performed to ensure that the stomach remained within the abdominal cavity (**Fig. 5**). A standard gastrostomy was created on the anterior wall of the gastric body and fixed to the abdominal wall approximately 2 cm caudal to the left costal margin. The stomach was secured to the abdominal wall with 5 interrupted 3-0 VICRYL sutures (Ethicon, Raritan, NJ, USA), and a 16-Fr gastrostomy button (MIC-KEY; Avanos Medical, Alpharetta, GA, USA) was placed. The gastrostomy was intended to provide both enteral access and fixation of the stomach within the abdominal cavity. No additional gastropexy sutures were placed.

**Fig. 4:**
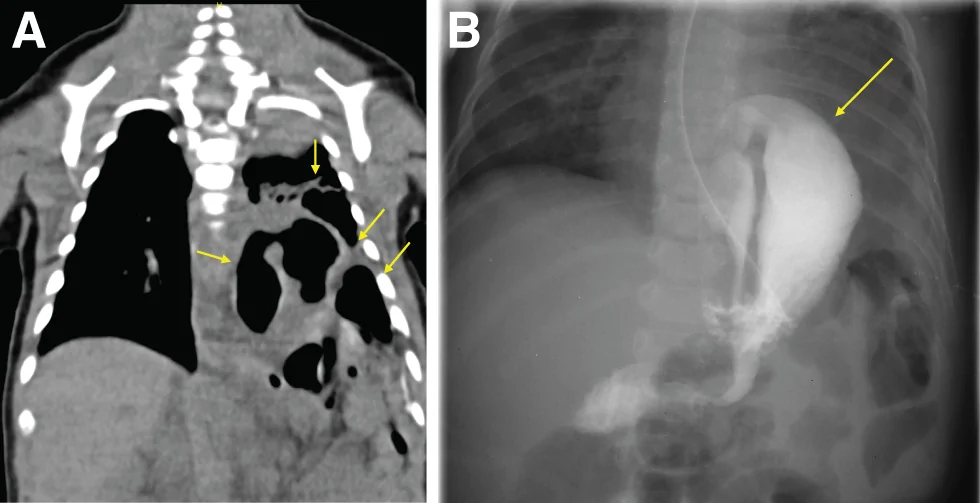
CT and upper gastrointestinal contrast study obtained at the time of subsequent recurrence. (**A**) Coronal CT image showing herniation of the stomach and bowel into the thoracic cavity (arrows). (**B**) Upper gastrointestinal contrast study demonstrating marked gastric herniation with mesenteroaxial volvulus (arrow).

**Fig. 5:**
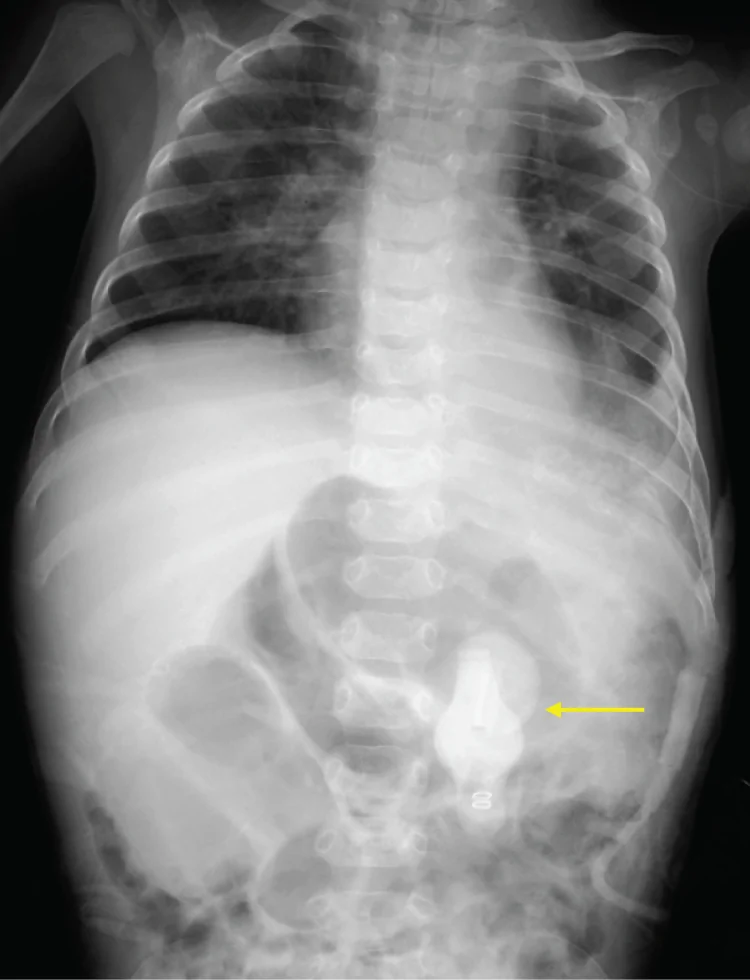
Radiograph showing the stomach in the abdominal cavity following additional diaphragmatic reconstruction and gastrostomy fixation. The arrow indicates the gastrostomy button.

No gastrostomy-related complications occurred. Because the patient achieved adequate oral intake and satisfactory growth without using the gastrostomy, the button was removed at 1 year of age. Persistent fixation of the stomach to the abdominal wall through the adhesion created by the gastrostomy was anticipated after button removal. At the most recent follow-up at 18 months of age, corresponding to 13 months after the final operation and 6 months after gastrostomy button removal, the patient demonstrated normal growth and development without evidence of CDH recurrence.

## DISCUSSION

Recurrence of CDH most commonly occurs at the lateral and posterior aspects of the repaired diaphragm.^2,9)^ Macchini et al.^2)^ suggested that recurrence tends to develop at the site of the original defect because most primary defects are located in the posterolateral diaphragm. In contrast, the anteromedial diaphragm is often preserved, allowing direct closure or secure patch fixation. In the present case, however, there was no medial diaphragmatic rim around the esophagus or mediastinum. Although the parietal pleura remained in this area, it was too thin to provide secure anchoring tissue. Consequently, the medial edge of the patch could only be placed over the defect without suturing. At both operations for recurrence, the lateral fixation remained intact, whereas the medial, anterior, and posterior portions had detached and the patch was displaced laterally. These findings suggest that the absence of secure medial fixation was an important anatomical and technical limitation of the reconstruction, although the exact sequence of patch detachment could not be determined.

Regarding the patch configuration, a flat, oversized patch might have represented a viable alternative to the cone-shaped patch. However, a flat patch would not have resolved the fundamental absence of reliable anchoring tissue around the esophagus and mediastinum. The cone-shaped patch configuration was selected to achieve tension-free reconstruction and increase the available abdominal capacity. However, because the medial overlap remained unsecured, we cannot exclude the possibility that the patch configuration and fixation method contributed to the vulnerability of the reconstruction.

Other potential configurations include a keyhole- or slit-shaped patch placed around the esophagus. However, there is a lack of evidence regarding the use of these patch configurations in infants with CDH. Although secondary hiatal hernia has been recognized as a complication after CDH repair,^9)^ no pediatric study has established the safety of placing a prosthetic patch circumferentially around the esophagus. In adult hiatal hernia repair, the placement of prosthetic mesh near or around the esophagus has been associated with dysphagia, fibrosis, stenosis, and erosion.^10)^ However, these complications have not been shown to be specific to the keyhole patch configuration, and the applicability of adult data to infants with CDH remains uncertain. Moreover, in the present patient, there was no sufficiently robust periesophageal or mediastinal tissue available to anchor the medial portion of such a patch. We therefore considered that a keyhole- or slit-shaped configuration would not have resolved the lack of reliable medial fixation and might have introduced additional risks of injury in the area around the esophagus.

Patients with CDH are also predisposed to gastric volvulus and splenic torsion because of abnormal fixation of the stomach and spleen.^11–13)^ Under normal circumstances, the stomach is stabilized by its ligamentous attachments, the esophageal hiatus, and the retroperitoneal fixation of the duodenum. However, in CDH, intrathoracic herniation of the abdominal viscera may be associated with incomplete development or laxity of the gastric ligaments, defects of the esophageal hiatus, and weakness of retroperitoneal fixation. In addition, insufficient formation or laxity of the gastrosplenic ligament has been implicated in the pathogenesis of both gastric volvulus and wandering spleen.^13)^

Miyauchi et al.^14)^ reported a patient with recurrent CDH who underwent transthoracic repair with direct closure and patch reinforcement but subsequently developed a peri-esophageal hiatal hernia requiring gastrostomy fixation. This previous report suggests that gastric fixation may play a role as an adjunctive procedure in selected patients with medial diaphragmatic deficiency or fragile periesophageal supporting structures^14)^; however, its effectiveness in preventing recurrence has not been established.

Oyachi et al.^15)^ reported a patient who developed recurrent splenic torsion and organoaxial gastric volvulus after CDH repair and was successfully treated with gastropexy. The authors speculated that inadequate fixation of the abdominal organs, combined with a tight primary repair, prevented the stomach and spleen from assuming their normal anatomical positions.^15)^ In our patient, the stomach repeatedly herniated into the thoracic cavity while exhibiting mesenteroaxial gastric volvulus. However, there is no clear causal relationship between gastric migration and the failure of the diaphragmatic reconstruction. The absence of reliable medial anchoring tissue and the unsecured medial overlap may have allowed initial separation of the patch and subsequent herniation of the stomach. Conversely, abnormal gastric mobility and repeated intrathoracic migration, particularly in association with gastric volvulus, may have imposed additional mechanical stress on the vulnerable reconstruction and promoted progressive patch detachment. These 2 mechanisms may have interacted; however, their sequence and relative contributions cannot be determined from this single case.

In the present patient, additional diaphragmatic reconstruction and gastrostomy fixation were performed simultaneously. Therefore, the respective contributions of these procedures to the favorable outcome cannot be determined. However, gastrostomy may have functioned not only as a route for enteral access but also as an adjunctive gastropexy by limiting gastric mobility. This combined approach may be considered in selected patients with recurrent CDH, medial diaphragmatic deficiency, and repeated gastric herniation.

## CONCLUSIONS

In this patient with recurrent CDH, medial diaphragmatic deficiency, and repeated gastric herniation, gastrostomy fixation was used as an adjunct to additional diaphragmatic reconstruction. Although its independent effect cannot be determined, fixation of the stomach may have contributed to limiting gastric mobility and reducing the likelihood of further herniation. This combined approach may be considered in selected patients with similar anatomical and clinical features.

## DECLARATIONS

### Availability of data and materials

The datasets supporting the conclusions of this article are included within the article.
